# Gewalt in intimen Partnerschaften und psychische Probleme bei Kindern und Jugendlichen: Online-Survey während der COVID-19-Pandemie

**DOI:** 10.1007/s00278-021-00501-w

**Published:** 2021-03-18

**Authors:** Vera Clemens, Franziska Köhler-Dauner, Ferdinand Keller, Ute Ziegenhain, Jörg M. Fegert, Michael Kölch

**Affiliations:** 1 Klinik für Kinder- und Jugendpsychiatrie/Psychotherapie, Universitätsklinikum Ulm, Steinhövelstr. 5, 89073 Ulm, Deutschland; 2 Klinik für Psychiatrie, Neurologie, Psychosomatik und Psychotherapie im Kindes- und Jugendalter, Universitätsmedizin Rostock, Rostock, Deutschland

**Keywords:** CoVid-19-Pandemie, Intime Partnergewalt, Psychische Probleme, Kinder und Jugendliche, Belastende Kindheitserlebnisse (ACEs), CoVid-19 pandemic, Intimate partner violence, Mental health problems, Children and adolescents, Stressful childhood experiences (ACEs)

## Abstract

**Hintergrund:**

Die durch die „coronavirus disease 2019“ (COVID-19) ausgelöste Pandemie hat das Leben von Familien in beispielloser Weise verändert. Während des ersten Lockdowns wurden außerfamiliäre Kontakte erheblich reduziert. Viele Eltern mussten parallel ihre Kinder betreuen und von zu Hause aus arbeiten, während der ökonomische Druck zunahm.

**Ziel der Arbeit (Fragestellung):**

Das Ziel der vorliegenden Studie war es, den Zusammenhang von Gewalt in intimen Partnerschaften und psychischen Problemen bei Kindern und Jugendlichen vor und während der COVID-19-Pandemie zu untersuchen.

**Material und Methoden:**

Mithilfe einer Online-Querschnittsumfrage wurden die Daten von 687 Eltern minderjähriger Kinder in Deutschland erhoben. Demografische und psychosoziale Prädiktoren für Gewalt in intimen Partnerschaften und Assoziationen mit psychischen Gesundheitsproblemen von Kindern vor, während und nach dem ersten Lockdown aufgrund der COVID-19-Pandemie wurden analysiert. Die Umfrage fand vom 18.05.2020 bis zum 21.07.2020 statt. Der Lockdown in Deutschland begann am 23.03.2020 und endete über schrittweise Lockerungen – die ersten Schulen öffneten am 22.04.2020 wieder; die Öffnungen von Schulen, Kindergärten und Kitas zog sich bis Ende Juni 2020.

**Ergebnisse:**

Ein geringeres Haushaltseinkommen und das Erleben von eigenen belastenden Kindheitserfahrungen erhöhen das Risiko, dass Studienteilnehmende Gewalt in ihrer Beziehung erfahren haben und darüber berichten. Kinder und Jugendliche, die in Familien leben, in denen Gewalt in intimen Partnerschaften vorkommt, wiesen nach Angaben der teilnehmenden Eltern vor und während der Pandemie häufiger höhere Werte für externalisierende Probleme auf, hinsichtlich emotionaler Probleme zeigten sich keine signifikanten Unterschiede.

**Diskussion:**

Belastende Kindheitserlebnisse erhöhen das Risiko für Gewalt in intimen Partnerschaften – und diese wiederum das Risiko für psychische Probleme der eigenen Kinder. Insofern sollte auch in der psychotherapeutischen Praxis systematisch nach entsprechenden Erfahrungen gefragt und entsprechende Behandlungsangebote sollte empfohlen werden.

## Abstract (en)

**Background:**

The coronavirus disease 2019 (COVID-19) pandemic has changed the lives of families in an unprecedented way. During the first lockdown, extrafamilial contact was significantly reduced. Many parents had to look after their children and work from home while the economic pressure increased.

**Objective:**

The aim of the present study was to investigate the association between intimate partner violence (IPV) and mental health problems in children and adolescents before and during the COVID-19 pandemic.

**Material and methods:**

In a cross-sectional online survey 687 parents of underaged children in Germany were included. Demographic and psychosocial predictors for IPV and associations with mental health problems of children before and during the first lockdown due to the COVID-19 pandemic were assessed. The survey ran from 18 May 2020 to 21 July 2020 and the lockdown in Germany began on 23 March 2020 and ended via a gradual relaxation. The first schools reopened on 22 April and the opening of schools, kindergartens and daycare centers extended to the end of June 2020.

**Results:**

Lower household incomes and the experience of own adverse childhood experiences increased the risk of participants reporting violence in their relationship. According to the participating parents, children and adolescents living in families in which violence was reported by intimate partners were more likely to have higher levels of externalizing problems before and during the pandemic. There were no significant differences with respect to emotional problems.

**Conclusion:**

Adverse childhood experiences increase the risk of intimate partner violence, which in turn increases the risk of mental health problems in the children. Therefore, in the psychotherapeutic practice questions should also be systematically asked about corresponding experiences and appropriate treatment offers should be recommended.

Belastende Kindheitserlebnisse („adverse childhood experiences“, ACE) erhöhen das Risiko für Gewalt in intimen Partnerschaften und diese wiederum das Risiko für psychische Probleme der eigenen Kinder. Es besteht die berechtigte Sorge, dass sich ein vorliegendes Risiko für das Erleben von häuslicher Gewalt in Familien aufgrund der verschlechterten Lebensbedingungen im Rahmen der durch die „coronavirus disease 2019“ (COVID-19) ausgelösten Pandemie verwirklicht bzw. sich bereits ausgelebte Gewalt verstärkt. Die vorliegende Untersuchung ist gemäß dem Wissen der Autoren eine der ersten, die sich dieses Themas unmittelbar während der Lockdown-Maßnahmen in Deutschland angenommen hat.

## Hintergrund und Fragestellung

Unter häuslicher Gewalt wird körperliche, aber auch psychische, sexuelle und wirtschaftliche Gewalt im Nahraum eines Haushalts verstanden, englisch als „domestic violence“ oder auch als „intimate partner violence“ bezeichnet, wobei sich letzterer Begriff spezifischer auf die Gewalt zwischen Erwachsenen in einer Partnerschaft bezieht (u. a. Sigurdsson 2019). Die Prävalenzangaben hängen von der Definition, der Gesetzeslage im jeweiligen Land, aber auch von sozialen und ökonomischen Faktoren ab (Gulati und Kelly 2020). Je nach Untersuchung werden Häufigkeiten von 14–45 % aller Frauen genannt, die Gewalt in intimen Partnerschaften erleben (Thompson et al. 2006, Sigurdsson 2019). Gewalt in intimen Partnerschaften kommt sowohl gegen Frauen wie auch gegen Männer vor, wobei Frauen häufiger Opfer werden als Männer.

Diese Gewalterfahrungen können Auswirkungen auf die psychische Gesundheit haben (Howard et al. 2010). Insofern spielt Gewalt in intimen Partnerschaften auch eine Rolle im psychotherapeutischen Kontext, sowohl in der Behandlung von (Folge‑)Störungen als auch ggf. als Raum, in dem Gewalterfahrungen erstmals thematisiert werden. Hierzu ist zu differenzieren, zwischen länger zurückliegenden Erfahrungen, auch Erfahrungen als Kind, und Gewalterfahrungen, die noch während einer Therapie stattfinden. Der psychotherapeutische Umgang ist dementsprechend unterschiedlich zu gestalten.

Eine Besonderheit ist, dass nicht allein das Erleben von Gewalt direkt gegen die eigene Person zu psychischen Folgen führen kann, sondern auch das Beobachten, etwa als Kind, wenn es zu Gewalt zwischen den Eltern kommt. Beim Auftreten von Gewalt in intimen Partnerschaften ist oft das gesamte familiäre System beeinträchtigt. So ist z. B. das Risiko für eine Beeinträchtigung der Eltern-Kind-Interaktionen bei Müttern, die häuslicher Gewalt ausgesetzt sind, erhöht (Levendosky et al. 2011). Dies erscheint vor dem Hintergrund der Belastungen durch Angst und Gewalt nicht verwunderlich. Die Kinder selbst kommen häufig in einen Loyalitätskonflikt. Ein Loyalitätskonflikt tritt z. B. auf, wenn das Kind versucht, Zuneigung und gute Gefühle gegenüber jedem seiner Elternteile aufrechtzuerhalten, obwohl diese gewalttätig zueinander sind. Dies kann zu großen Belastungen führen. Wendet das Kind sich hingegen mehr einem Elternteil zu, kann es Schuldgefühle gegenüber dem anderen Elternteil bekommen. Die Schuldgefühle werden noch verstärkt, wenn die Elternteile jeweils die emotionale Zustimmung des Kindes erwarten oder sogar direkt einfordern.

Auch das Erleben anderer Kindheitsbelastungen, zu denen Gewalt in intimen Partnerschaften als eine Form, aber zudem auch noch weitere Formen von Haushaltsdysfunktionen und Kindesmisshandlung zählen (Felitti et al. 1998), erhöht das Risiko für potenziell schädliche Erziehungsmaßnahmen (Clemens et al. 2020). Insbesondere das Erleben mehrerer Arten von Kindheitsbelastungen („adverse childhood experiences“, ACE) steigert das Risiko für körperliche und psychische Probleme im Erwachsenenalter (Felitti et al. 1998; Clemens et al. 2018b; Riedl et al. 2020).

Metaanalysen zu diesem Thema zeigen, dass sowohl das Risiko für internalisierende Störungen, wie Depression, als auch für externalisierende Störungen, wie z. B. aggressive Verhaltensweisen im Rahmen einer Störung des Sozialverhaltens, wuchs, wenn in der Kindheit Gewalt zwischen den Eltern miterlebt wurde (Kitzmann et al. 2003; Wolfe et al. 2003). Die Risiken für soziale Probleme und schlechtere Schulergebnisse sind bei Kindern und Jugendlichen, die Gewalt zwischen den Eltern erleben, ebenfalls erhöht (Kitzmann et al. 2003). Ebenso konnte eine gesteigerte Bereitschaft für riskante Verhaltensweisen, einschließlich Drogenkonsum (Bair-Merritt et al. 2006) und Alkoholmissbrauch (Caetano et al. 2003), bei betroffenen Kindern/Jugendlichen nachgewiesen werden.

Das Miterleben von Gewalt zwischen den Eltern ist häufig. In einer Untersuchung an einer repräsentativen Stichprobe der deutschen Bevölkerung gaben kürzlich knapp 10 % der Befragten an, in der Kindheit Gewalt gegen die Mutter oder Stiefmutter miterlebt zu haben (Witt et al. 2019).

Durch die COVID-19-Pandemie hat sich das Leben von Familien in beispielloser Weise verändert (s. auch den Beitrag von Reis et al. in diesem Heft). Schätzungsweise 90 % der Kinder und Jugendlichen waren zwischenzeitlich von Schulschließungen betroffen (UNESCO 2020). Soziale Kontakte außerhalb der eigenen Kernfamilie wurden stark eingeschränkt, viele Freizeitaktivitäten verboten (Team 2020). Eltern waren oft einer doppelten Belastung ausgesetzt: Sie mussten Kinder betreuen und ältere Kinder/Jugendliche beim Homeschooling unterstützen, während sie parallel dazu im Homeoffice arbeiteten. Der finanzielle Druck hat in vielen Familien aufgrund von Einkommenseinbußen zugenommen. Auch wirtschaftliche Probleme können zu Stress und konsekutiv zu Ehekonflikten und einer Zunahme häuslicher Gewalt führen (Elder 1974; Elder und Conger 2000; Stith et al. 2004). Berichte über häusliche Gewalt und Anrufe bei spezialisierten Beratungsangeboten haben während des ersten Lockdowns im Frühling 2020 in vielen Ländern, auch in Deutschland, zugenommen (UN women 2020).

Das Ziel der vorliegenden Studie war es daher, den Zusammenhang von Gewalt in intimen Partnerschaften und psychischen Problemen bei Kindern und Jugendlichen vor und während der COVID-19-Pandemie zu untersuchen. Auf Basis der dargestellten Literatur lauteten die Hypothesen, dass

- erhöhter wirtschaftlicher Druck, geringere Bildung und das Erleben von ACE Risikofaktoren für Gewalt in intimen Partnerschaften darstellen und
- Kinder aus Familien, in denen Gewalt zwischen den Eltern vorkommt, mehr psychische Probleme aufweisen, und dass diese Probleme während des Lockdowns stärker zugenommen haben als bei Kindern aus Familien ohne Gewalt zwischen den Eltern.

## Studiendesign und Untersuchungsmethoden

### Stichprobe und Prozedere

Die Daten wurden über eine Online-Umfrage erhoben, die vom 18.05.2020 bis zum 21.07.2020 stattfand. Die Umfrage erfolgte über die Plattform unipark. Informationen über die Umfrage wurden über die Homepage der Uniklinik Ulm, soziale Medien und Printmedien sowie über bestehende Mailing-Listen von anderen Studien verbreitet.

Bevor Interessierte zu der eigentlichen Umfrage gelangten, wurden Informationen über die Studie und die Datenanalyse gegeben. Alle Teilnehmenden mussten vor der Teilnahme in eine elektronische Teilnahmeerklärung einwilligen. Die Teilnahme war anonym. Die Teilnehmenden konnten die Umfrage jederzeit ohne Angabe von Gründen beenden. Einschlusskriterien waren die Einwilligung in die Teilnahme und der Abschluss des 18. Lebensjahres. Insgesamt begannen 1826 Teilnehmende die Studie, 1405 (76,94 %) sendeten den Fragebogen zurück. In der vorliegenden Untersuchung wurden nur die Daten der Teilnehmenden ausgewertet, die den Fragebogen abschlossen und die angaben, minderjährige Kinder zu haben (*n* = 687).

Die Studie wurde in Übereinstimmung mit den ethischen Richtlinien durchgeführt, die in der Erklärung von Helsinki von 1964 festgelegt wurden. Nach Rücksprache mit der Ethikkommission der Universität Ulm bestand aufgrund des anonymen Charakters der Umfrage keine Notwendigkeit für ein Ethikvotum.

### Instrumente

Zu den soziodemografischen Daten, die erhoben wurden, gehörten Alter, Geschlecht, Bildungsniveau, das subjektive Auskommen mit dem Haushaltseinkommen, Gewalt in der Beziehung und Einkommensveränderungen während der Pandemie. Belastende Kindheitserfahrungen der Erwachsenen wurden mithilfe der deutschen Version des Fragebogens „Adverse Childhood Experiences Questionnaire“ (ACE Questionnaire; Felitti et al. 1998) erfasst, eines Standardinstruments zur retrospektiven Erhebung belastender Kindheitserfahrungen mit zufriedenstellender interner Konsistenz (Cronbachs α = 0,76; Wingenfeld et al. 2011).

Die psychischen Probleme der Kinder wurden mithilfe einzelner Items aus der deutschen Elternversion des „Strengths and Difficulties Questionnaire“ (SDQ) erhoben (Woerner et al. 2002), eines Screeninginstruments für Verhaltensauffälligkeiten bei Kindern und Jugendlichen (Goodman 1997). Um die Länge der Befragung zu begrenzen, erfolgte die Auswahl der Items selektiv. Folgende Items wurden genutzt: „unruhig, überaktiv, kann nicht lange stillsitzen“, „klagt häufig über Kopfschmerzen, Bauchschmerzen oder Übelkeit“, „ständig zappelig“, „oft unglücklich oder niedergeschlagen; weint häufig“, „hat viele Ängste; fürchtet sich leicht“, „hat oft Wutanfälle; ist aufbrausend“, „hat viele Sorgen; erscheint häufig bedrückt“, „verhält sich gegenüber Erwachsenen oft widerwillig“ und „nervös oder anklammernd in neuen Situationen; verliert leicht das Selbstvertrauen“.

### Analyse der Daten

Die statistischen Analysen wurden mit SPSS, Version 21, durchgeführt. Die Prädiktoren für das Auftreten häuslicher Gewalt in der Beziehung wurden mithilfe einer logistischen Regression berechnet. Anhand von T‑Tests wurden die psychischen Probleme von Kindern und Jugendlichen in Familien mit und ohne häusliche Gewalt jeweils vor und während der Pandemie verglichen.

## Ergebnisse

### Stichprobe

Die Teilnehmenden waren überwiegend Frauen (*n* = 614, 89,5 %). Das mittlere Alter der Teilnehmerinnen betrug 41,4 Jahre (Standardabweichung [SD] ± 7,4 Jahre, Alterspanne 26 bis 67 Jahre), das der Teilnehmer 45,8 Jahre (SD ± 8,0 Jahre, Altersspanne 33 bis 71 Jahre). Die Mehrheit der männlichen sowie weiblichen Teilnehmenden lebte mit einer*m Partner*in zusammen (w: *n* = 518, 84,2 %; m: *n* = 65, 90,3 %). Der Bildungsgrad war hoch, die Mehrheit hatte als höchsten Abschluss das Abitur oder einen Fachhochschul- bzw. Universitätsabschluss (w: *n* = 416, 67,6 %; m: *n* = 56, 77,8 %). Eine Minderheit war finanziell so von der Pandemie betroffen, dass das Einkommen um mehr als ein Viertel reduziert war (w: *n* = 69, 11,2 %; m: *n* = 6, 8,3 %). Die demografischen Daten sind in Tab. 1 zusammengefasst.

**Table 1:** 

	Frauen	Männer	*p-*Wert
*Anzahl der Teilnehmenden, n* (%)	615 (89,5)	72 (10,5)	–
*Alter, M* *(±* *SD*;* Jahre)*	41,4 (± 7,4)	45,8 (± 8,0)	<0,001
*Alterspanne (Jahre)*	26–67	33–71	–
*Höchster akademischer Abschluss, n* (%)			
Universitätsabschluss oder (Fach‑) Abitur	416 (67,6)	56 (77,8)	–
Anderer oder kein Schulabschluss	199 (32,4)	16 (22,2)	0,079
*Einkommensrückgang >* *25* *% seit der Pandemie, n* (%)	69 (11,2)	6 (8,3)	0,455
*Auskommen mit dem Haushaltseinkommen, n* (%)			
Teilnehmende kommen gut aus	384 (62,4)	44 (61,1)	–
Teilnehmende kommen aus	163 (26,3)	21 (29,2)	–
Teilnehmende kommen gerade so aus	51 (8,3)	7 (9,7)	–
Teilnehmende kommen kaum aus	18 (2,9)	0 (0,0)	0,480
*Gewalt in der Partnerschaft, n* (%)	34 (6,1)	2 (2,9)	0,413
*Anzahl belastender Kindheitserfahrungen, M* *(±* *SD*;* Jahre)*	1,7 (± 1,9)	1,3 (± 1,4)	0,011

*M* Mittelwert, *SD* Standardabweichung

### Prädiktoren für häusliche Gewalt

Teilnehmende, die mit ihrem Haushaltseinkommen nach eigener Einschätzung kaum auskamen, hatten ein signifikant höheres Risiko, häusliche Gewalt zu erfahren als Teilnehmende, die berichteten, mit ihrem Haushaltseinkommen auszukommen („odds ratio“ [OR] = 9,58, *p* < 0,01). Je mehr Formen belastender Kindheitserfahrungen von den Teilnehmenden in der Kindheit erlebt wurden, desto höher war ihr Risiko, von Gewalt in ihrer Beziehung betroffen zu sein (OR = 1,21, *p* < 0,05). Geschlecht, Lebensalter, Bildungsabschluss und Einbußen im Einkommen seit Beginn der Pandemie stellten keine signifikanten Prädiktoren für häusliche Gewalt in der untersuchten Stichprobe dar (Tab. 2).

**Table 2:** 

	χ^2^ (df)	*p*-Wert	R^2^	Odds ratio	95 %-KI	*p-*Wert
**Modell 1**	1,328 (2)	0,515	0,006	–	–	–
Männliches Geschlecht	–	–	–	0,473	0,109–2,049	0,317
Alter (Jahre)	–	–	–	0,997	0,950–1,047	0,905
**Modell 2**	3,298 (3)	0,348	0,015	–	–	–
Männliches Geschlecht	–	–	–	0,497	0,115–2,156	0,350
Alter (Jahre)	–	–	–	0,997	0,950–1,046	0,899
Universitätsabschluss oder (Fach‑)Abitur	–	–	–	0,604	0,302–1,209	0,154
**Modell 3**	11,956 (5)	0,035	0,054	–	–	–
Männliches Geschlecht	–	–	–	0,535	0,122–2,337	0,405
Alter (Jahre)	–	–	–	1,008	0,959–1,059	0,755
Universitätsabschluss oder (Fach‑)Abitur	–	–	–	0,545	0,268–1,109	0,094
Einkommenssenkung um > 25 %	–	–	–	1,772	0,484–6,489	0,388
Teilnehmende kommen kaum mit dem Haushalteinkommen aus	–	–	–	11,823	2,457–56,901	0,002
**Modell 4**	16,266 (6)	0,012	0,074	–	–	–
Männliches Geschlecht	–	–	–	0,597	0,136–2,624	0,495
Alter (Jahre)	–	–	–	1,004	0,955–1,056	0,866
Universitätsabschluss oder (Fach‑)Abitur	–	–	–	0,608	0,295–1,252	0,177
Einkommenssenkung um > 25 %	–	–	–	1,935	0,521–7,191	0,324
Teilnehmende kommen kaum mit dem Haushalteinkommen aus	–	–	–	9,584	1,957–46,940	0,005
ACE-Summenscore	–	–	–	1,205	1,017–1,427	0,031

Prädiktoren für häusliche Gewalt, berechnet mithilfe einer binär logistischen Regressionsanalyse, *n* = 617

*ACE* „adverse childhood experiences“

### Psychische Probleme in Abhängigkeit von häuslicher Gewalt in der Familie

**Figure 1:**
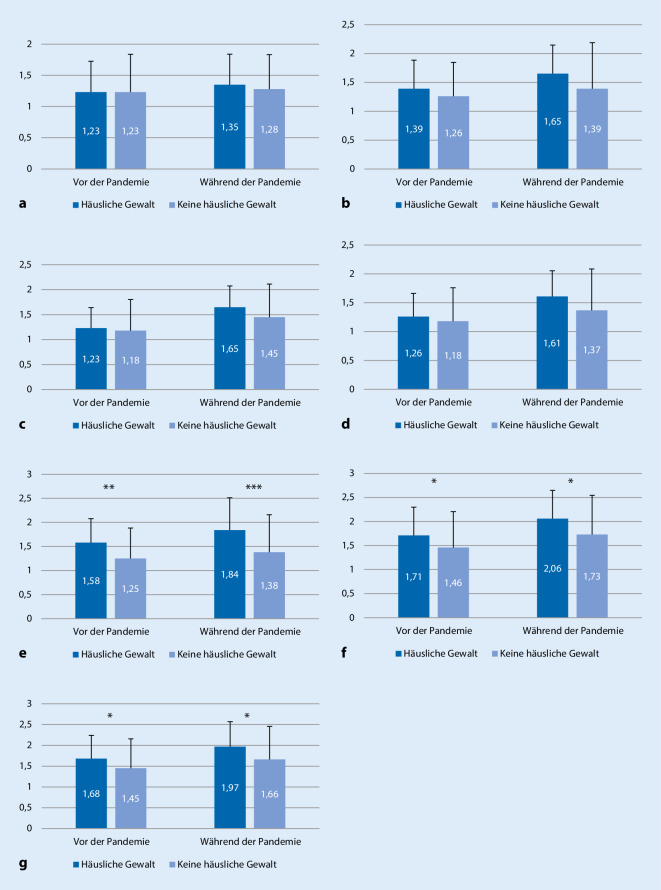

Im Vergleich zur Zeit vor der Pandemie nahmen alle problematischen Verhaltensweisen bei Kindern und Jugendlichen in Familien, in denen keine Gewalt unter den Eltern herrscht, nach Einschätzung der Eltern, signifikant zu (Unruhe: 1,36 vs. 1,53, *p* < 0,001, Schmerzen: 1,23 vs. 1,28, *p* = 0,034; zappelig: 1,25 vs. 1,38, *p* < 0,001; unglücklich/niedergeschlagen: 1,17 vs. 1,45, *p* < 0,001; Ängste: 1,26 vs. 1,39, *p* < 0,001; Wutanfälle: 1,46 vs. 1,73, *p* < 0,001; widerwillig: 1,45 vs. 1,66, *p* < 0,001; nervös/anklammernd: 1,31 vs. 1,43, *p* < 0,001). Bei den Kindern und Jugendlichen, in deren Familie Gewalt zwischen den Eltern herrschte, nahmen die problematischen Verhaltensweisen nach Einschätzung der Eltern ebenso zu, nicht signifikant war diese Zunahme lediglich bei den Items Schmerzen sowie nervös/anklammernd (Unruhe: 1,65 vs. 2,00, *p* = 0,006, Schmerzen: 1,23 vs. 1,35, *p* = 0,103; zappelig: 1,58 vs. 1,84, *p* = 0,018; unglücklich/niedergeschlagen: 1,23 vs. 1,65, *p* = 0,001; Ängste: 1,39 vs. 1,65, *p* = 0,030; Wutanfälle: 1,71 vs. 2,06, *p* = 0,006; widerwillig: 1,68 vs. 1,97, *p* = 0,010; nervös/anklammernd: 1,48 vs. 1,58, *p* = 0,264). Kinder und Jugendliche, die in Familien leben, in denen von häuslicher Gewalt berichtet wurde, waren nach Einschätzung der Eltern signifikant häufiger zappelig, bereits vor der Pandemie (t(31,965) = −2,669, *p* < 0,05) sowie während der Pandemie (t(555) = −3,870, *p* < 0,001). Kinder und Jugendliche in Familien mit Partnerschaftsgewalt zeigten zudem signifikant häufiger Wutausbrüche, sowohl vor (t(551) = −2,302, *p* < 0,05) als auch während der Pandemie (t(554) = −2,449, *p* < 0,05). Ein signifikanter Unterschied in Abhängigkeit davon, ob zwischen den Eltern Gewalt berichtet wurde, fand sich des Weiteren dafür, ob Kinder/Jugendliche oft widerwillig gegenüber Erwachsenen sind. Der Mittelwert war sowohl vor (t(554) = −2,177, *p* < 0,05) als auch während der Pandemie (t(554) = −2,345, *p* < 0,05) signifikant höher als bei Kindern/Jugendlichen, aus deren Familien keine häusliche Gewalt berichtet wurde. Hinsichtlich emotionaler Probleme stellte sich folgender Trend dar: Emotionale Probleme sind bei Kindern und Jugendlichen, in deren Familien Gewalt zwischen den Eltern vorkommt, stärker ausgeprägt, und zudem werden die Unterschiede während der Pandemie eher größer. Eine statistische Signifikanz wurde hier jedoch nicht gefunden (Abb. 1).

## Diskussion

Inzwischen werden vermehrt Studienergebnisse zu möglichen psychischen Folgen als Auswirkung der COVID-19-Pandemie bzw. der entsprechenden Maßnahmen zur ihrer Eindämmung publiziert (u. a. Gulati und Kelly 2020; Lai et al. 2020; Torales et al. 2020; Vindegaard und Benros 2020). Hinsichtlich der häuslichen Situation gab es auch in Deutschland die Sorge, dass es in Familien, in denen das Risiko für das Erleben von Gewalt erhöht ist, ggf. zu einer Zunahme häuslicher Gewalt kommen könnte (Fegert et al. 2020). Generell können sowohl psychische Probleme bei Eltern als auch kindliche Psychopathologie ein Risikofaktor dafür sein, dass Vernachlässigung und Misshandlung vorkommen (Clemens et al. 2018a). Die vorliegende Untersuchung ist gemäß dem Wissen eine der ersten, die unmittelbar während der Lockdown-Maßnahmen in Deutschland Eltern im Zusammenhang mit Vorerfahrung eigener ACE und dem kindlichen problematischen Verhalten befragt hat.

Im Laufe der Untersuchung nahmen die psychischen Probleme bei Kindern und Jugendlichen zu. Diese Erkenntnis stimmt mit anderen Ergebnissen überein (Ravens-Sieberer et al. 2020) und weist auf die Belastung von Kindern und Jugendlichen durch die Pandemie sowie durch die dadurch bedingten Maßnahmen, wie Schulschließungen, Wegfall von außerfamiliären Kontakten, hin. Generell ist aber festzuhalten, dass es sich nicht um klinische Diagnosen, sondern um die dimensionale Ausprägung in einem Screeningverfahren handelt. Die Untersuchung zeigt, dass psychische Probleme sowohl vor als auch während der Pandemie stärker bei Kindern und Jugendlichen, in deren Familien Gewalt zwischen den Eltern vorkommt, ausgeprägt sind als bei nicht von Gewalt in intimen Partnerschaften betroffenen Kindern und Jugendlichen. Diese Unterschiede traten insbesondere bei externalisierenden Symptomen zutage, während bezüglich internalisierender Probleme in der untersuchten Stichprobe weder vor noch während der Pandemie signifikante Unterschiede in Abhängigkeit vom Auftreten häuslicher Gewalt in der Familie festgestellt wurden. Dies mag daran liegen, dass internalisierende Störungen generell von Eltern weniger gut wahrgenommen werden als externalisierende Störungen, insbesondere wenn Eltern selbst belastet sind (Los Reyes und Kazdin 2005; Kelley et al. 2017). Der Befund verdeutlicht umso mehr, dass spezielle Gruppen, die bereits vor der Pandemie besonderen Risiken ausgesetzt waren, dies auch während der Pandemie sind.

Eine Untersuchung zur Rolle von Gewalt in intimen Partnerschaften zeigte, dass diese und psychische Misshandlung sich auf psychische Langzeitfolgen, wie Depressionen und Angstzustände in Kombination, stärker auswirken als sexueller Missbrauch durch Familienmitglieder oder Bekannte (Teicher et al. 2006). Es scheint also, dass nicht nur „klassische“ Misshandlungsformen wie z. B. sexueller Missbrauch, sondern auch Exposition gegenüber häuslicher und psychischer Gewalt – Misshandlungsformen, die häufig als weniger „schlimm“ erachtet werden – die kindliche Entwicklung maßgeblich schädigen können (Clemens et al. 2018b, 2020). Insofern weisen die erhobenen Daten darauf hin, dass es vermutlich einer stärkeren adjustierten Risikoeinschätzung dazu bedarf, welche Personen ggf. auch unter Pandemiebedingungen der Unterstützung bedürfen. Kinder und Jugendliche, die in Haushalten mit Gewalt zwischen den Eltern aufwachsen, würden hierzu gehören.

Zudem legen Studienergebnisse nahe, dass ein Zusammenhang existiert, zwischen in der Kindheit miterlebter Gewalt unter den Eltern und dem Risiko, dass Söhne im Erwachsenenalter selbst Täter sowie Töchter Opfer häuslicher Gewalt werden (Wood und Sommers 2011). Diese Ergebnisse weisen auf eine intergenerationelle Übertragung von Gewalt in intimen Partnerschaften hin und verdeutlichen die Notwendigkeit, Kinder zu schützen, um diesen „Teufelskreis der Gewalt“ zu unterbrechen.

Ein weiterer Risikofaktor für das Auftreten von Gewalt in intimen Partnerschaften bestand, wenn das Haushaltseinkommen kaum ausreichte. Dieses Ergebnis stimmt mit anderen Untersuchungen überein, die zeigen, dass wirtschaftliche Probleme mit dem Auftreten häuslicher Gewalt assoziiert sind (Elder 1974; Elder und Conger 2000; Stith et al. 2004). Inzwischen liegen auch diesbezügliche Publikationen im Zusammenhang mit der COVID-Pandemie vor (Sharma und Borah 2020; Bryant et al. 2020). Auch eine Reduktion des Einkommens war mit einem erhöhten Risiko für Partnerschaftsgewalt verbunden. Hier muss als Limitation der Studie benannt werden, dass die Stichprobe ein sehr hohes Bildungsniveau aufwies und zudem die Einschätzung des Haushaltseinkommens rein subjektiv erfolgte. Interessanterweise scheint es jedoch v. a. die subjektive Erwartung der wirtschaftlichen Bedrohung sein, die das Risiko für intrafamiliäre Gewalt während Rezessionen steigert (Lee et al. 2013; Schneider et al. 2017). Allerdings ist hervorzuheben, dass nur 7 % der Varianz durch dieses Modell erklärt werden können. Es ist es also anzunehmen, dass noch weitere wichtige Risikofaktoren, die im Rahmen der Umfrage nicht erhoben wurden, eine Rolle spielen. Aus der Literatur bekannte Faktoren sind u. a. psychische Erkrankungen und Substanzabusus beim Partner/bei der Partnerin, kultureller Hintergrund, soziale Unterstützung und Stress (Capaldi et al. 2012).

## Limitationen der Studie

Die Ergebnisse der vorliegenden Untersuchung unterstreichen bereits bekannte Befunde aus der Wissenschaft auch für diese deutsche Stichprobe vor und während des ersten Lockdowns. Dennoch sind einige Limitationen hervorzuheben. Die Ergebnisse beruhen nicht auf der Umfrage einer repräsentativen Stichprobe. Die Angaben zu psychischen Problemen bei Kindern und Jugendlichen basieren auf Antworten der Eltern. Hierbei können insbesondere internalisierende Probleme unterschätzt worden sein. Zudem handelt es sich um eine Querschnittserhebung. Die retrospektive Erfassung von psychischen Problemen, aber auch Kindheitsbelastungen kann zu Verzerrungen führen. Auch weitere familiäre Faktoren, wie z. B. Zahl, Geschlecht und Alter der Kinder, könnten Einfluss auf die untersuchten Zusammenhänge haben und zu Verzerrungen führen. Da Gewalt in intimen Partnerschaften häufig mit anderen Formen von Kindesmisshandlungen einhergeht (Clemens et al. 2019), ist es wahrscheinlich, dass die betroffenen Kinder noch mehr Formen von ACE erlebt haben – was ebenso zu Verzerrungen führen kann.

## Fazit für die Praxis

- Die gezeigten Daten legen nahe, dass Gewalt in intimen Partnerschaften das Risiko für psychische Probleme erhöht – auch transgenerational. Kinder, die im häuslichen Kontext Gewalt miterleben, entwickeln mehr psychische Auffälligkeiten, als Kinder, die diese Erfahrungen nicht machen.
- In der psychotherapeutischen Praxis sollte systematisch nach entsprechenden Erfahrungen gefragt und ggf. sollten etwaige psychische Probleme der Kinder erhoben werden.
- Da externalisierendes Verhalten von Kindern wiederum im Zusammenhang mit dem ungünstigen Erziehungsverhalten von Eltern steht, kann dieses die Gefahr für häusliche Gewalt auch gegen Kinder erhöhen.
- Bei Hinweisen auf das Vorkommen von häuslicher Gewalt und kindlichen psychischen Auffälligkeiten sind entsprechende Behandlungsangebote auch für das Kind zu empfehlen.
